# Editorial: Insights in obstetric and pediatric pharmacology: 2021

**DOI:** 10.3389/fphar.2022.995923

**Published:** 2022-09-14

**Authors:** Jeffrey S. Barrett

**Affiliations:** Aridhia Digital Research Environment, Glasgow, United Kingdom

**Keywords:** obstetrics, pediatrics, innovation, translational research, collaboaration

Recent years have provided unprecedented opportunities to advance translational science in obstetric and pediatric pharmacology. With our Insights 2021 Research Topic presented herein we also appreciate that there is still much to do. Previous works have pointed out some of the challenges in our domain; many of these relate to the still conservative nature of clinical research in obstetric and pediatric populations and sub-populations (Blehar et al., 2013; Grimsrud et al., 2015) but also include technologies that have yet to expand to accommodate the unique nuances that define these populations (Wancura et al., 2019; Scientific and Technical Advisory Council, 2020) as well as the gaps in physiologic data that needs to be first generated and then shared among the relevant stakeholders (Moya et al., 2014; Chan et al., 2021) in our ecosystem. Fifteen fantastic papers have been accepted in this thematic topic covering a multitude of issues but most highlighting the knowledge gaps that remain and the continued effort required to fill these gaps. Highlighting these submissions are papers that address advances in dried blood spot (DBS) analytical methodologies and their utility in supporting material and fetal sampling in pregnancy studies, physiologically-based PK (PBPK) and population-based PK (PPK) modeling analyses to assess various pregnancy sub-population (e.g., obese pregnant women and pregnant women receiving drug therapy), gene and cell therapy for pediatric patients, and drug metabolism knowledge gaps in pregnant and pediatric populations.

An encouraging theme among these papers is that they not only that they address the aforementioned knowledge gaps, but they all represent building blocks by which future preclinical and clinical investigations in obstetric and pediatric pharmacology can proceed. DBS assays have been around for a long time with many illustrating the benefit in the clinical conduct in obstetric and pediatric clinical trials and still there is a reluctance to invest in the approach prospectively to support new drug research and development due to concerns about transitivity and cross-validation with traditional analytical methodologies and regulatory acceptance (Amini et al., 2021; Blázquez-Gamero et al., 2021). Likewise, PBPK has long been appreciated as a relevant approach to facilitate the design and analysis of obstetric and pediatric pharmacology clinical trials, but few examples have been generated to spur more widespread use and adoption (Gaohua et al., 2012; Zhang et al., 2017) though more recent investigations suggest that their incorporation into clinical development plans improve both dosing guidance and drug monograph labeling (Coppola et al., 2021; Gill and Jones, 2022). Beyond PBPK, artificial intelligence and machine learning approaches are now being bought to bear in drug development more commonly and regulatory experience is growing with more expanded applications (Liu et al., 2022). Koch et al. remind us that there are opportunities for these newer quantitative approaches to be applied in perinatology, providing an excellent application in the treatment of jaundice in preterm and term neonates.

Therapeutic proteins still represent a gap as a modality for pediatrics and pregnant women though new promising agents in this class provide an opportunity to fill these gaps in addition to benefit patients.

Their development however typically requires some manner of bridging from small molecule experience in these populations. Meibohm paper highlights the knowledge gaps and prioritizes efforts to reduce them for the purpose of accelerating new TP development in these at-risk populations.

Cell and gene therapy for obstetric and pediatric pharmacology populations continues to be an important therapeutic modality requiring additional study. Several critical therapeutic areas including cancer, ischemia, and several rare diseases (Gupta and Anderson, 2014) all represent Frontier areas where translational research is needed for both risk assessment and definition of the therapeutic window for agents under investigation. Currently, there are 3 FDA-approved gene therapies for pediatric patients, but more are in various stages of research and development. Challenges remain including those related to recruiting and educating patients, capital incentives that support the R&D investment, and financial considerations onwhether and how reimbursement of such therapies will be provided. Hence, beyond the drug development challenges, there are concerns about how to appropriately value and benefit from these modalities.

These papers are both timely and clinically relevant in their own right, but they are also likely to be foundational for future work to come. Obstacles remain for obstetric and pediatric pharmacology investigation. Table 1 highlights some of the more commonly identified barriers. Most concerning about the table is that many of the items listed have been identified for some time prompting more serious discussion regarding how we as a community make more substantive progress in some of the core areas centered around education, data sharing, and collaboration. Some progress has been made in fact so we can be encouraged. Recent efforts to support data sharing and collaboration in the rare disease ecosystem bodes well for many of the topics highlighted herein (Larkindale et al., 2022) and recent NICHD grants supporting the collaboration of research projects for obstetric and pediatric patient populations encourage both multidisciplinary collaboration, education, and data sharing (Pawlyk, 2022). Specifically, The Obstetric and Pediatric Pharmacology and Therapeutics Branch of NICHD has established the MPRINT Hub to aggregate, present, and expand the available knowledge, tools, and expertise in maternal and pediatric therapeutics to the broader research, regulatory science, and drug development communities. It will serve as a national resource for conducting and fostering therapeutics-focused research in obstetrics, lactation, and pediatrics while enhancing the inclusion of people with disabilities. Our hope with this Research Topic is that there exists a diverse community of multidisciplinary scientists dedicated to reducing gaps and improving the landscape of obstacles that prohibit progress.

**TABLE 1:** Biggest obstacles for obstetric and pediatric pharmacology investigation.

Obstacle	Current State
Empiric dosing practices	While some pediatric centers of excellence are embracing more modern dosing practices including more quantitative approaches to individualized dosing, the default is still empiric-driven at most in-patient settings and certainly the case for outpatients
Lack of creative, collaborative, multidisciplinary research approaches	Some positive examples exist but they are typically confined to a few therapeutic areas (e.g., oncology)
Reliance on adult drug development experience for dosing, efficacy, and safety expectations for these populations	Clinical development plans for both obstetric and pediatric populations are almost nonexistent unless the target indication is specifically for one or both groups; otherwise plans focus on bridging strategies heavily reliant on the adult (non-pregnant) patient experience
Limited consideration of indications that exist only in neonates or during pregnancy	Financial incentives to develop these indications largely do not exist limiting prioritization from would-be developers
Lack of biomarkers for these populations	Obviously linked to other factors but despite a well-recognized deficit remains lacking
Lagging pharmacoepidemiologic investigations	Access to data challenging; less incentives for planning and operational resources needed
Better data bridges to inform decision making	Access and connectivity of data streams not in place; concerns regarding IP and the typical bottlenecks around data sharing

## References

[B1] Amini F. Auma E. Hsia Y. Bilton S. Hall T. Ramkhelawon L. (2021). Reliability of dried blood spot (DBS) cards in antibody measurement: A systematic review. PLoS ONE 16 (3), e0248218. 10.1371/journal.pone.0248218 33720928 PMC7959368

[B2] Blázquez-Gamero D. Sánchez B. Folgueira M. D. (2021). Dried blood spot testing for detection of congenital cytomegalovirus. JAMA Pediatr. 175 (8), 865–866. 10.1001/jamapediatrics.2021.0755 33938947

[B3] Blehar M. C. Spong C. Grady C. Goldkind S. F. Sahin L. Clayton J. A. (2013). Enrolling pregnant women: Issues in clinical research. Womens Health Issues 23 (1), e39–45. 10.1016/j.whi.2012.10.003 23312713 PMC3547525

[B4] Chan G. J. Daniel J. Getnet M. Kennedy M. Olowojesiku R. Hunegnaw B. M. (2021). Gaps in maternal, newborn, and child health research: A scoping review of 72 years in Ethiopia. J. Glob. Health Rep. 5, e2021033. 10.29392/001c.22125

[B5] Coppola P. Kerwash E. Cole S. (2021). Physiologically based pharmacokinetics model in pregnancy: A regulatory perspective on model evaluation. Front. Pediatr. 9, 687978. 10.3389/fped.2021.687978 34249817 PMC8262675

[B6] Gaohua L. Abduljalil K. Jamei M. Johnson T. N. Rostami-Hodjegan A. (2012). A pregnancy physiologically based pharmacokinetic (p-PBPK) model for disposition of drugs metabolized by CYP1A2, CYP2D6 and CYP3A4. Br. J. Clin. Pharmacol. 74 (5), 873–885. 10.1111/j.1365-2125.2012.04363.x 22725721 PMC3495152

[B7] Gill K. L. Jones H. M. (2022). Opportunities and challenges for PBPK model of mAbs in paediatrics and pregnancy. AAPS J. 24, 72. 10.1208/s12248-022-00722-0 35650328

[B8] Grimsrud K. N. Sherwin C. M. Constance J. E. Tak C. Zuppa A. F. Spigarelli M. G. (2015). Special population considerations and regulatory affairs for clinical research. Clin. Res. Regul. Aff. 32 (2), 47–56. 10.3109/10601333.2015.1001900 26401094 PMC4577021

[B9] Gupta A. Anderson S. (2014). Cell and gene therapy: Overview, current landscape and future trends. J. Precis. Med. 11. https://www.thejournalofprecisionmedicine.com/the-journal-of- precision-medicine/cell-and-gene-therapy-overview-current-landscape-and-future-trends/.

[B10] Larkindale J. Betourne A. Borens A. Boulanger V. Theurer Crider V. Gavin P. (2022). Innovations in therapy development for rare diseases through the rare disease cures accelerator-data and analytics platform. Ther. Innov. Regul. Sci. 56, 768–776. 10.1007/s43441-022-00408-x 35668316

[B11] Liu Q. Huang R. Hsieh J. Zhu H. Tiwari M. Liu G. (2022). Landscape analysis of the application of artificial intelligence and machine learning in regulatory submissions for drug development from 2016 to 2021. Clin. Pharmacol. Ther. 2022, 16. Epub ahead of print. PMID: 35707940. 10.1002/cpt.2668 35707940

[B12] Moya J. Phillips L. Sanford J. Wooton M. Gregg A. Schuda L. (2014). A review of physiological and behavioral changes during pregnancy and lactation: Potential exposure factors and data gaps. J. Expo. Sci. Environ. Epidemiol. 24 (5), 449–458. 10.1038/jes.2013.92 24424408

[B13] Pawlyk A. (2022). Maternal and pediatric precision in therapeutics (MPRINT) Hub, NIH, obstetric and pediatric pharmacology and therapeutics Branch. https://www.nichd.nih.gov/about/org/der/branches/opptb/mprint#.

[B14] Scientific and technical advisory Council (STAC) of the special journals publisher (SJP): Research design innovations in obstetrics, gynecology, and pediatrics. Special J. Obstetrics, Gynecol. Pediatr. [SJ-OGPAPR], 2020; 1 (1):1–21.

[B15] Wancura M. McCracken J. M. Steen E. Cosgriff-Hernandez E. Keswani S. Hakim J. C. (2019). Emerging technologies in pediatric gynecology: New paradigms in women's health care. Curr. Opin. Obstet. Gynecol. 31 (5), 309–316. 10.1097/GCO.0000000000000563 31369479 PMC8428278

[B16] Zhang Z. Imperial M. Z. Patilea-Vrana G. I. Wedagedera J. Gaohua L. Unadkat J. D. (2017). Development of a novel maternal-fetal physiologically based pharmacokinetic model I: Insights into factors that determine fetal drug exposure through simulations and sensitivity analyses. Drug Metab. Dispos. 45, 920–938. 10.1124/dmd.117.075192 28588050 PMC5506457

